# Pedestrian walking speed monitoring at street scale by an in-flight drone

**DOI:** 10.7717/peerj-cs.1226

**Published:** 2023-01-25

**Authors:** Dan Jiao, Teng Fei

**Affiliations:** School of Resource and Environmental Sciences, Wuhan University, Wuhan, Hubei, China

**Keywords:** Walking speed, UAV, Pedestrian identification, Pedestrian tracking

## Abstract

The walking speed of pedestrians is not only a reflection of one’s physiological condition and health status but also a key parameter in the evaluation of the service level of urban facilities and traffic engineering applications, which is important for urban design and planning. Currently, the three main ways to obtain walking speed are based on trails, wearable devices, and images. The first two cannot be popularized in larger open areas, while the image-based approach requires multiple cameras to cooperate in order to extract the walking speed of an entire street, which is costly. In this study, a method for extracting the pedestrian walking speed at a street scale from in-flight drone video is proposed. Pedestrians are detected and tracked by You Only Look Once version 5 (YOLOv5) and Simple Online and Realtime Tracking with a Deep Association Metric (DeepSORT) algorithms in the video taken from a flying unmanned aerial vehicle (UAV). The distance that pedestrians traveled related to the ground per fixed time interval is calculated using a combined algorithm of Scale-Invariant Feature Transform (SIFT) and random sample consensus (RANSAC) followed by a geometric correction algorithm. Compared to ground truth values, it shows that 90.5% of the corrected walking speed predictions have an absolute error of less than 0.1 m/s. Overall, the method we have proposed is accurate and feasible. A particular advantage of this method is the ability to accurately predict the walking speed of pedestrians without keeping the flight speed of the UAV constant, facilitating accurate measurements by non-specialist technicians. In addition, because of the unrestricted flight range of the UAV, the method can be applied to the entire scale of the street, which assists in a better understanding of how the settings and layouts of urban affect people’s behavior.

## Introduction

Walking speed is known as the sixth vital sign (Fritz & Lusardi, 2009), besides heart rate, respiratory rate, blood pressure, body temperature and pain. It is the core parameter of the urban pedestrian micro-model (Willis et al., 2004), which has important research significance for urban planning, pedestrian traffic safety and public health, *etc*. In the field of public health, researchers have studied the correlations between walking speed and other factors such as a pedestrian’s gender, age (real age and subjective age) and health status (Tolea et al., 2010; Nolan et al., 2018; Stephan, Sutin & Terracciano, 2015) using a controlled variable method. Using this approach, authors have used walking speeds to assess the general health of a population (Middleton, Fritz & Lusardi, 2015) and to predict the risk of mortality (Lusardi, 2012). In the area of urban planning, good pedestrian facilities can encourage residents to maintain environmentally friendly travel patterns, and can enhance street vibrancy. A large body of literature has used walking speed as an important indicator (Rastogi, Thaniarasu & Chandra, 2011; Silva, da Cunha & da Silva, 2014; Al-Azzawi & Raeside, 2007; Chen, Zhao & Shi, 2016) to reflect the level of service of facilities. On the other hand, unlike previous ways of assessing street walkability based on objective indicators, walking speed can be used to reflect pedestrians’ willingness to stay and move around on the street. It provides feedback on the pedestrian’s experience of the environment from the perspective of the street user, which helps urban planners to make a more comprehensive walkability assessment. In addition, pedestrian speed, as a key input for various traffic engineering applications (Montufar et al., 2007), can assist in the design of carriageways (Gore et al., 2020) and street crossings (Chandra & Bharti, 2013). Measuring the walking speeds of special groups such as elderly people can also have benefits in terms of adjusting the timing of traffic signals and reducing traffic fatalities (Duim, Lebrão & Antunes, 2017).

Following recent developments in science and technology, methods of acquiring walking speeds have changed. Initially, a stopwatch was used to calculate walking speed by recording the time it took for a walker to pass a marker (Finnis & Walton, 2008; Montero-Odasso et al., 2020; Youdas et al., 2006; Oh et al., 2019), but this method requires large number of measurers, and is inefficient. To overcome the disadvantages of the field observation method, new acquisition methods based on images, trails, and wearable devices have been developed (MejiaCruz et al., 2021). However, these methods also have their own drawbacks. The most common technique for trail-based methods is the timing gate (van Loo et al., 2003; Martin et al., 2019; Kong & Chua, 2014; Warden et al., 2019), which requires the walker to wear a sensor chip, but walking speed is significantly affected when experimental participants are aware that they are being observed (Obuchi, Kawai & Murakawa, 2020), and the installation of the timing gate also requires extensive hardware investment. Image-based acquisition is a more efficient and practical data acquisition method (Hussein et al., 2015; Liang et al., 2020; Sikandar et al., 2021; Franěk & Režnỳ, 2021; Hediyeh et al., 2014), but requires multiple cameras in different locations for image acquisition over a large geographical area, and the cost and technical requirements are high due to the collaboration and placement of multiple cameras. If only a single camera is used to collect data, the image-based acquisition method, like the previous two methods, can only extract walking speeds over a very small area. Although wearable devices do not need to be limited to fixed road sections (Cha et al., 2017; Morey et al., 2017; Silsupadol, Teja & Lugade, 2017), the measurement of walking speeds through cell phone location data compromises the user’s personal privacy, and it is difficult for experimental participants to cooperate with the experiment over a long period, resulting in a low return rate of data and a high overall cost.

Unmanned aerial vehicles (UAVs) have efficient scene capture capabilities, and have gained popularity in fields such as digital forensics (Bouafif et al., 2018), potentially dangerous event analysis (Rădescu & Dragu, 2019), and parcel delivery (Mekala & Baig, 2019). The camera is easy to place and operate, and the device can easily be moved to hover in locations where video surveillance cannot be installed (Yeom & Cho, 2019). Furthermore, a UAV acquires data without interfering with pedestrians, and the cost and technical requirements are not high. Hence, it is widely used for the acquisition of mobile pedestrian video sequence data (Fang & Kim, 2020; Bian et al., 2016; Chang et al., 2018; Kim, 2020; Miyazato, Uehara & Nagayama, 2019), as it is not subject to the shortcomings of the above methods. After acquiring video data, the key step in extracting walking speed is to perform pedestrian detection and tracking. This is an intensely researched problem in computer vision: detection involves determining whether a pedestrian is present in an image or video, while the main task of tracking is to associate the most similar pedestrians in the previous and subsequent frames (Wang, Sun & Li, 2019). Deep learning has now become mainstream, due to its good feature extraction ability (Xiao et al., 2021; Li, Wu & Zhang, 2016; Saeidi & Ahmadi, 2018). This method allows for continuous tracking of a large number of pedestrians using a multi-objective tracker, which facilitates the analysis of individual or group behaviour patterns (Cao, Sai & Lu, 2020). It has been experimentally demonstrated that nearly 1,000 people can be continuously and dynamically identified using deep learning (Xue & Ju, 2021), and the accuracy may be greater than 0.95, even for a pedestrian density of 9.0 per m^2^ (Jin et al., 2021).

In view of the advantages of UAVs and deep learning, we used a UAV to obtain videos of pedestrians on city streets, and detected and tracked each pedestrian using You Only Look Once (YOLO), a representative model of convolutional neural networks, detected ground movement due to drone flight using the Scale-Invariant Feature Transform (SIFT) and random sample consensus (RANSAC) algorithms, and finally extracted the walking speeds of the pedestrians on the streets.

## Study area

**Figure 1:**
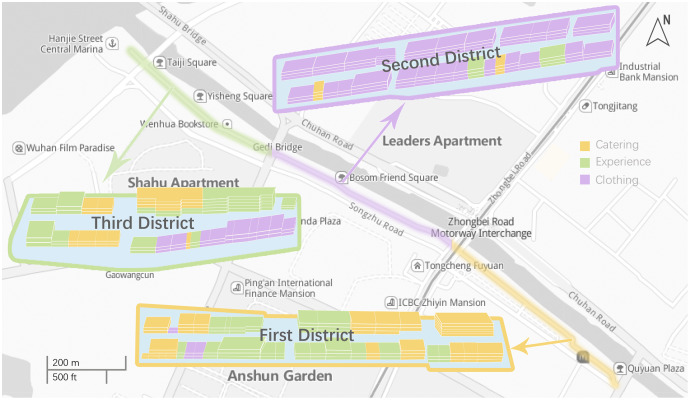
The study area Hanjie Street.

The commercial pedestrian street of Chuhe Hanjie, located in Wuhan, China, forms part of Wuhan’s central cultural district. With a total length of 1.5 km, it is currently the longest urban commercial pedestrian street in China. According to data provided by the Wuhan Anti-epidemic applet, the average cumulative pedestrian traffic on Hanjie Street can reach 0.1 million in a single day. As shown in Fig. 1, Hanjie Street has three districts with more than 200 domestic and foreign merchants, including shopping, food, culture, leisure, entertainment and other types, and clear planning based on functional zoning. In addition to commercial stores, Hanjie Street also hosts a popular theatre and several small squares. There are no trees along the entire length of the street on both sides, which not only provides good views for window shopping by pedestrians, but also allows us to extract pedestrian walking speeds from videos captured by a UAV.

## Method

### Experimental design

**Figure 2:**
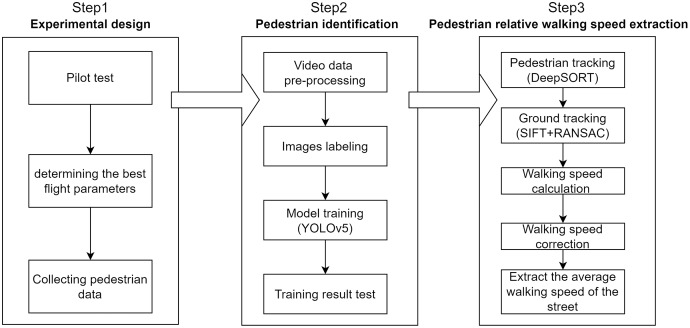
Flowchart for our study.

As shown in Fig. 2, our experiment involved three steps: experimental design, pedestrian identification, and extraction of relative pedestrian walking speeds. At the experimental design stage, two pilot tests were carried out to obtain the optimal flight height and speed. The environmental conditions and sensor parameters used for data collection are also explained in this section. Pedestrian detection was performed using You Only Look Once Version 5 (YOLOv5). In the step involving the extraction of relative walking speeds, pedestrian tracking was performed using an algorithm called Simple Online and Realtime Tracking with a Deep Association Metric (DeepSORT). The SIFT and RANSAC algorithms were employed to achieve ground tracking. The pedestrian speeds were then calculated and the error in the speeds caused by image point displacement were corrected.

#### Pilot tests

The image resolution of videos acquired by a UAV varies at different flight altitudes, and the accuracy of pedestrian recognition and walking speed extraction will be affected as a result. It is therefore necessary to find the optimal altitude at which both a high pedestrian recognition rate and a large field of view can be obtained. The flight speed of the drone is also a key factor affecting the accuracy of the walking speed calculations. Too low a speed may result in a limited efficiency, while too high a speed may result in insufficient clarity of the frames of the video recording. Pilot tests are therefore needed to determine the optimal height and speed of the UAV before extracting street-scale pedestrian walking speeds.

The site used for the pilot tests met the following requirements: (i) it was a relatively open area, without coverings such as trees; (ii) there were enough pedestrians passing by to give a suitable sample size; (iii) it was paved with tiles of the same size, which enabled the true values of the walking speeds of volunteers to be calculated based on the number of tiles crossed per second.

After finding a suitable experimental site, the pilot tests were carried out in two parts. Firstly, to explore the optimal flight height for the UAV, videos of pedestrians walking were collected from an overhead view at different heights while the UAV was hovering. Since the heights of most buildings were lower than 40 m, three heights of 40, 60 and 80 m were chosen as experimental heights. In the second part, to determine an appropriate horizontal flight speed for the UAV, videos of pedestrians walking were collected at different UAV speeds and for three different pedestrian walking states (standing still, slow walking, and fast walking) at the optimal height. Seven different flight speeds (0, 1, 2, 3, 4, 5, and 6 m/s) were used to find the optimal flight speed of the UAV for each walking state.

#### Collecting pedestrian data

**Figure 3:**
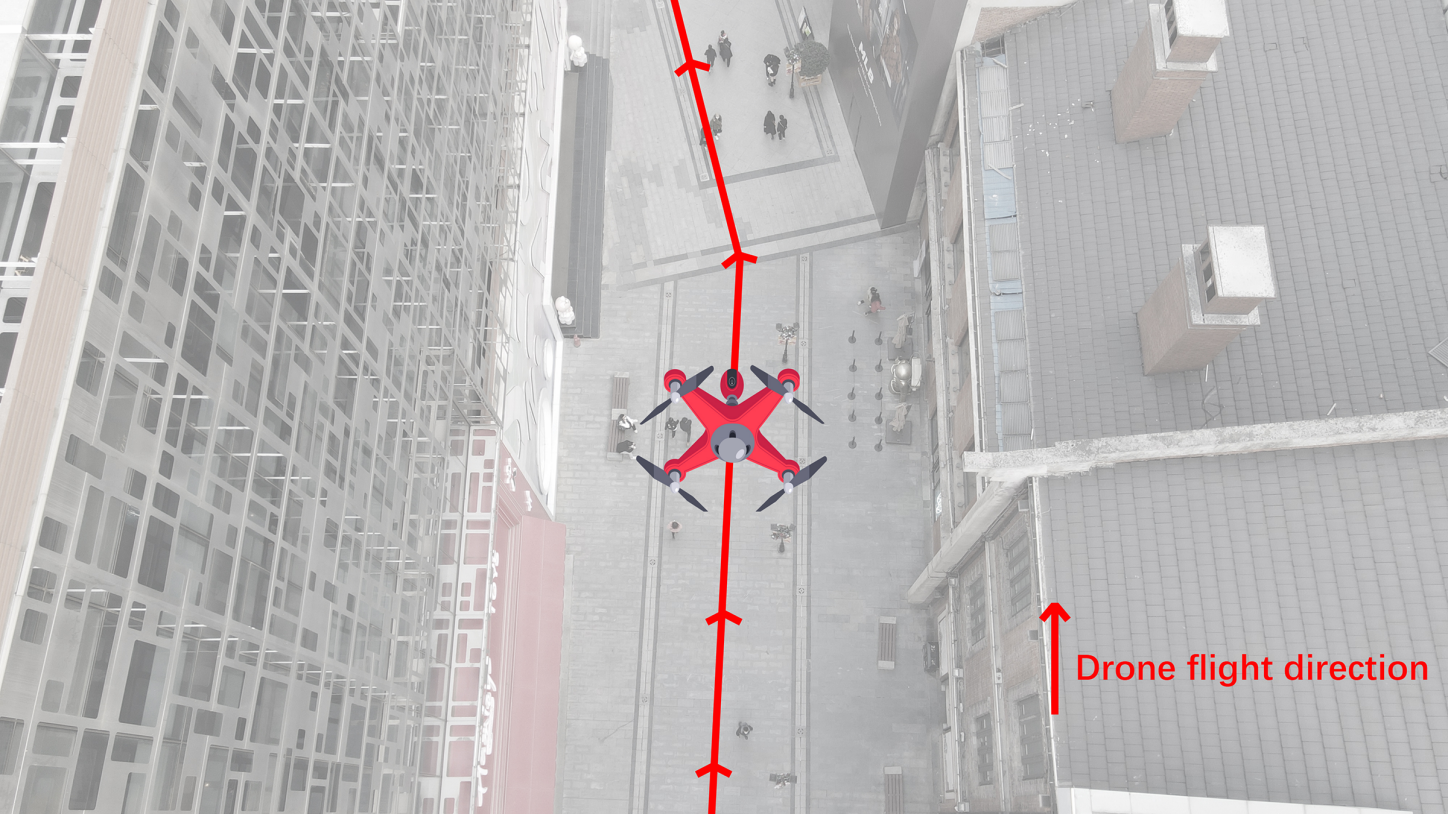
Flight heading along the direction of the street.

A sunny weekday afternoon (from 3–5 pm) was chosen for the collection of pedestrian data, and an open location at the end of the street was selected for take-off. When the drone had reached the optimal flight altitude, we rotated the drone lens through 90° and adjusted it to an overhead view angle, always flying forward along the direction of the street, as shown in Fig. 3. During the flight, the drone maintained a selected constant speed, and the white balance and ISO were kept automatic. In addition, considering that the UAV needs to change batteries midway to maintain flight, the data are collected in segments according to the functional partition of the study area and stored in MP4 mode with the resolution set to 1,920 × 1,080 p.

#### Sensor parameters

In this experiment, we used a Mavic Air 2 to acquire the pedestrian walking videos. This is a small, consumer-grade, rotary-wing UAV with a Sony IMX586 camera, 48 million effective pixels, a maximum flight take-off height of 5,000 m, and a maximum horizontal flight speed of 19 m/s. It is lightweight and flexible, weighs only 570 g, and the wings are foldable. It also has low site requirements, and can hover at a fixed point for up to 33 min (in an environment without wind). The key parameters of the drone are shown in Table 1.

**Table 1:** Basic parameters of the Mavic Air 2.

Image sensor	1/2 CMOS
Field of view	84°
Focal length	24 mm (35 mm isometric)
Photo resolution	8,000 × 6,000
Video resolution	FHD: 1,920 × 1,080 p

### Pedestrian identification

In order to perform pedestrian tracking from videos, pedestrians must first be detected in single images. YOLO is a state-of-the-art, real-time object detection algorithm that allows the user to manually find a trade-off between speed and accuracy (Redmon et al., 2016; Lan et al., 2018). The new YOLOv5 (Jocher, 2020) algorithm, proposed in 2020, provides data enhancement through the use of data loaders for scaling, colour space adjustment and mosaic enhancement, and is able to automatically learn the size of the anchor frame, which makes it suitable for the detection of small targets. It also runs relatively fast and flexibly, while maintaining accuracy. This algorithm was therefore selected to achieve pedestrian detection.

Since the image dataset obtained in this experiment was small, the training, test and validation sets were defined based on a ratio of 6:2:2.

#### Video data pre-processing

Before performing pedestrian detection, we removed footage containing few pedestrians and serious tilting or swinging of the UAV, and then converted the remaining video to images at a frame rate of 30 fps.

Due to the images are all from a high transmission frame rate conversion of the video, resulting in hundreds of images that are similar and also increasing the likelihood of overfitting the training model. Therefore, we randomly selected some converted images to form the training dataset to avoid unnecessary duplication. There are three parts of experiment: two pilots and walking speed extraction in the study area. Accordingly, we obtained three images datasets called “altitude” (720 images), “flight speed” (1,890 images), and “street” (1,300 images), all of which were captured on a weekday afternoon in December and were stored in PNG format.

The “altitude” dataset contained 240 images at each of three altitudes (40, 60 and 80 m). The “flight speed” dataset contained 90 images for each of the seven flight speeds for each of the three pedestrian states. The “street” dataset consisted of images of pedestrians drawn from nine video clips, including two clips captured from the first district (with a total of 389 images), three clips from the second district (611 images), and four clips from the third district (300 images).

#### Image labelling

Since the acquired dataset was captured directly, annotation of the data was required before training the images. The process of data annotation is a manual labelling process that provides machine systems with samples used for learning. By manually selecting the targets and marking the categories, allowing the computer to continuously learn the features of this data, ultimately enabling the computer to recognize it autonomously. An annotation tool called LabelImg was adopted to label the images of pedestrians. The final YOLOv5 label file contained five parameters per line: the object class, the centre coordinates of the detection object, and the width and height of the detection object.

The “altitude” dataset contained a total of 20,758 pedestrian labels, whereas the “flight speed” dataset had a total of 10,368. The “street” dataset had a total of 19,443 pedestrian labels, of which the training set had 780 images with 11,506 labels, the test set had 260 images with 3,970 labels, and the validation set had 260 images with 3,967 labels.

#### Model training and verification

The deep learning framework used in this experiment was PyTorch (GPU version 1.70), and the Jupyter notebook tool Colab (GPU parameters: Tesla P100, CUDA 11.2, RAM 25GB), provided by Google, was used to train the model. The weights file used for training in this experiment was YOLOv5x, which saves the weights of each layer of the network trained by the pre-trained set. YOLOv5x is pre-trained based on COCO dataset (a large, rich object detection dataset provided by the Microsoft team) and has the highest detection accuracy of the YOLOv5 models. The iteration batch size was set to 16, the initial learning rate was 0.01, and the momentum was set to 0.937. A total of 50 training rounds were completed, at which the loss tends to be stable and the model begins to converge, and the best training model was chosen for pedestrian detection.

As for verification, sufficient numbers of pedestrians (*N* > 100) for each of the three UAV altitudes (40, 60, and 80 m), and each of the seven flight speeds (0, 1, 2, 3, 4, 5, and 6 m/s) were randomly selected to test the pedestrian recognition rate. When the pedestrian recognition rate was 80% or more, the flight parameters were considered to satisfy the experimental requirements for extracting pedestrian walking speeds. The confidence threshold for detection was set to 0.15 (confidence levels below this value were not shown in the images), and the final detected image was marked with a rectangular box to indicate the recognized object class and confidence level.

### Relative walking speed of pedestrians

#### Pedestrian tracking

DeepSORT is a commonly used algorithm in multi-target tracking (Ciaparrone et al., 2020) that offers cascade matching and trajectory confirmation (unlike its predecessor algorithm SORT), which is beneficial for the short-term prediction of targets (Wojke, Bewley & Paulus, 2017). In addition, the most important feature of DeepSORT is the addition of an appearance feature description value, which can greatly improve the target ID transformation after a long period of occlusion. Thus, the DeepSORT algorithm is ideally suited for tracking moving targets in potentially obscured areas.

In this experiment, we used the best performing model from the previous model training as the weight file for YOLOv5, while the DeepSORT weight file used the default file obtained from pre-training based on the Market1501 dataset. The Market1501 dataset is collected from six cameras on the Tsinghua University campus, with a total of 1,501 pedestrians labelled, making it one of the most commonly used datasets in the field of pedestrian recognition. After inputting the original video, the DeepSORT algorithm first obtains the target detection frame by the target detector YOLOv5, and then predicts the trajectory of the pedestrian using Kalman filtering. Then features are extracted for the corresponding pedestrians in the target frames, and the match between the pedestrians in the before and later frames is calculated according to the Hungarian algorithm. Finally, each pedestrian in the image is assigned a different ID. In the pedestrian track file, each line contains six values, the first value indicates the frame in which the target appears, the second value indicates the ID number of the target and the third to sixth values are the coordinates and size of the bounding box. With the saved pedestrian track file, we can get the position of each pedestrian in each frame of the video.

#### Ground tracking

To calculate the relative walking speed of the pedestrians, two distances needed to be obtained: the distance of pedestrian movement in the image per fixed time interval, and the “ground movement” relative to the image coordinate in that period of time. As the movement of the UAV is affected by wind, an absolutely uniform speed without lateral movement could not be guaranteed. A method was therefore needed to obtain the “ground movement” in the non-hovering state. We first needed to obtain corresponding points in the previous and subsequent frames by feature matching, and then to calculate the single-response matrix based on these corresponding points.

In this experiment, we used a combination of the SIFT and RANSAC algorithms to extract the distance moved by the ground in the image. The SIFT algorithm is a region detection algorithm that was proposed by Lowe (1999) and further refined in 2004 (Lowe, 2004), which has high speed and local feature invariance. The opensource toolkit VLFeat was utilised to compute the SIFT features of the images. The angle between the vectors was used as the distance metric descriptor, and to enhance the robustness of matching, we performed bidirectional matching (in which we first computed the feature matching values from the latter image to the former image, and then the feature matching values from the former to the latter).

The corresponding image points in the previous and subsequent frames obtained by matching with the SIFT algorithm may contain both correct and false matches. However, the RANSAC algorithm can correctly estimate the parameters of a mathematical model by iteration from a set of data containing a large amount of noise (Fischler & Bolles, 1981; Derpanis, 2010), and was therefore applied to eliminate false matches, which improved the accuracy of the subsequent single-response matrix calculation. The parameters of the algorithm in the experiment were set as follows. The minimum number of data values required to fit the model was 50; the maximum number of iterations allowed in the algorithm was 5,000; the threshold value for determining when a data point fitted a model was 7e3 as the default; and the number of close data values required to assert that a model fitted the data well was 300.

#### Calculation of walking speeds

After using the DeepSORT algorithm to achieve pedestrian tracking, position information was acquired on the pedestrians in each frame. The single-response matrix obtained using the RANSAC algorithm after eliminating mismatched points was used to calculate the distance moved by the ground in the image. The real walking distance of the pedestrian could then be acquired, as shown in Eq. (1):

(1) $${S_2} = {S_1}{\rm \;-\ dis}\left\{ {{X_1},{X_2}} \right\}$$

where *S*_*2*_ is the real walking distance of the pedestrian, *S*_*1*_ is panning distance of the ground in the image, *X*_*1*_ is the position of the pedestrian in the previous frame, *X*_*2*_ is the position of the pedestrian in the subsequent frame, and dis{*X*_*1*_, *X*_*2*_} is the distance moved by the pedestrian in the image.

After determining the real distance moved by the pedestrian, the walking speed can be obtained as shown in Eq. (2):

(2) $${v_m}^\prime = {S_2}/n \cdot fps$$

where *v*_*m*_*′* is the pedestrian speed (in units of pixels/s at this point), *S*_*2*_ is the real walking distance of the pedestrian, *fps* is the frame rate, and *n* is the interval between the two selected frames.

The field-of-view diameter can be calculated as shown in Eq. (3):

(3) $$d = 2 \cdot H \cdot \tan (FOV/{\rm 2})$$

where *d* is the field-of-view diameter, *FOV* is the field-of-view angle, and *H* is the height of the UAV.

The size of the field of view can be calculated using Eq. (4):

(4) $$\eqalign{& L = \sqrt {{d^2}/({l^2} + {w^2})} \cdot l \cr & W = \sqrt {{d^2}/({l^2} + {w^2})} \cdot w}$$

where *L* and *W* are the length and width of the field of view, *d* is the field-of-view diameter, and *l: w* is the aspect ratio of the image.

The speed can then be calculated using Eq. (5):

(5) $${v_m} = L/M \cdot {v_m}^{\prime}$$

where *v*_*m*_ is the pedestrian speed (m/s), *L* is the length of the field of view, *M* is the length of the field of the image, and *v*_*m*_*′* is the pedestrian speed (in pixels/s).

**Figure 4:**
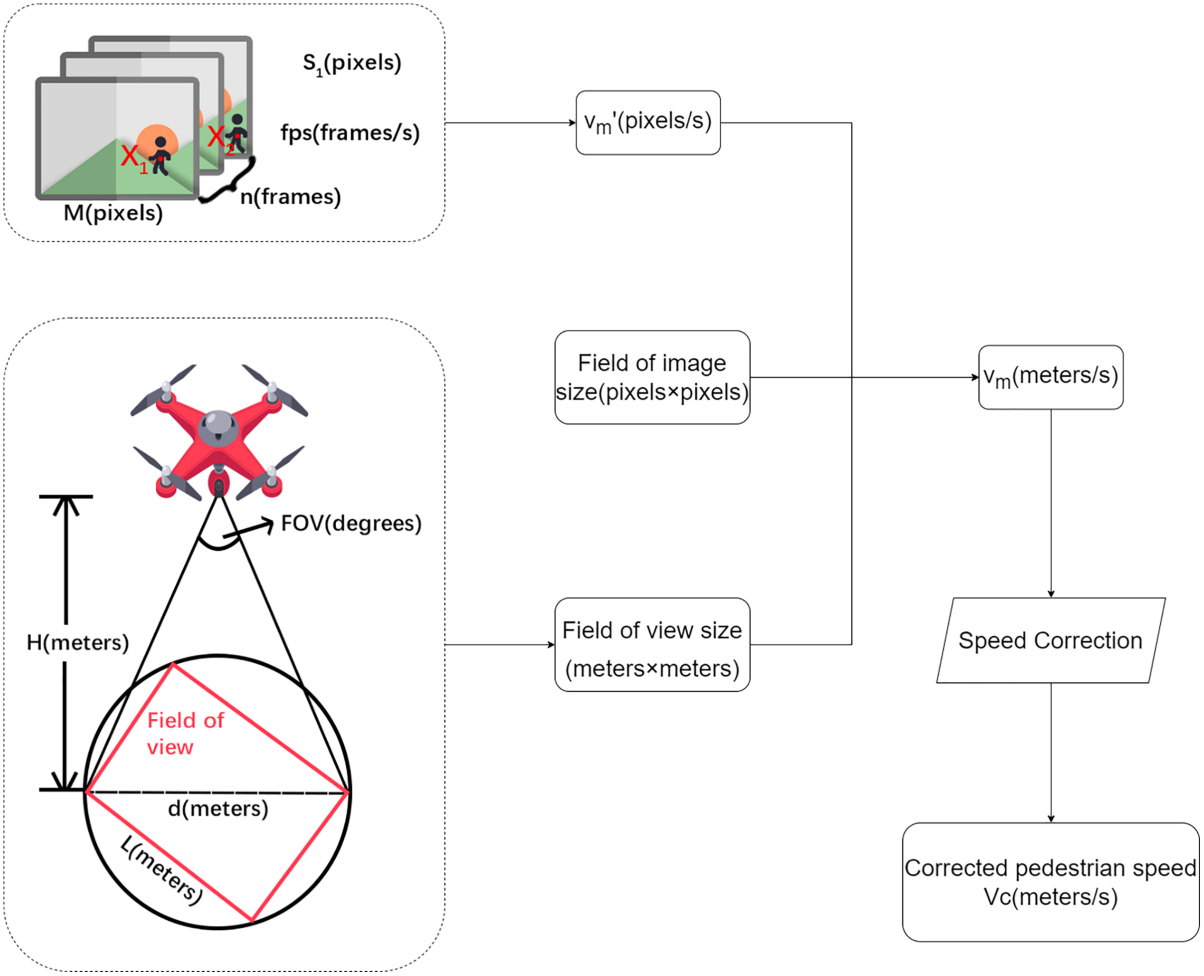
Flowchart for describing the calculation steps and variables relationship in between.

A Flowchart for describing the calculation steps and variables relationship in between is shown in Fig. 4.

#### Correction to walking speeds

Since the image taken by the UAV is in essence a central projection of the ground, while the YOLO5 locates the heads of pedestrian which are at a certain height of the ground. This introduces an geometric error to the walking distance measurement, and hence a geometric correction is needed to retrieve more accurate walking speed.

**Figure 5:**
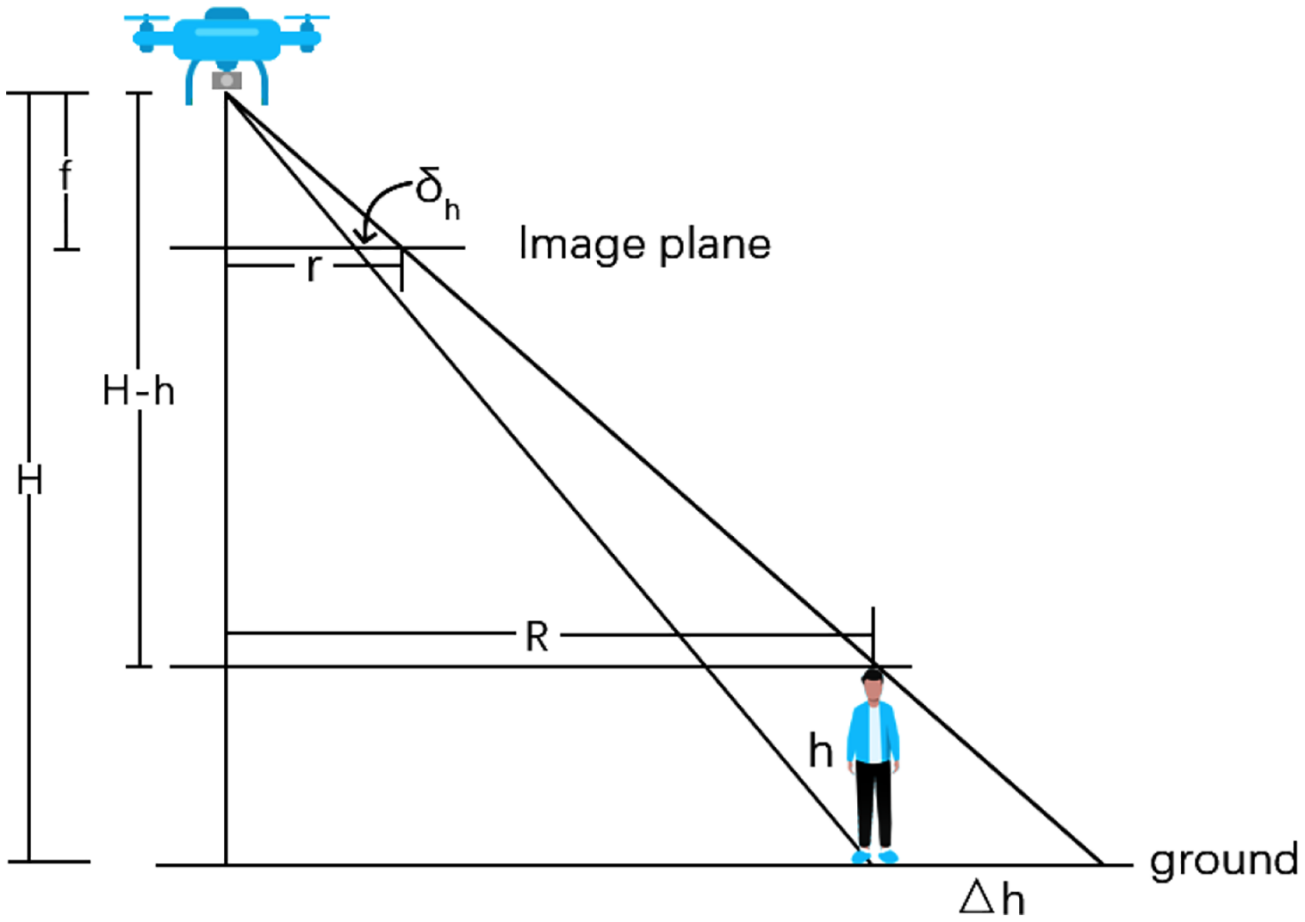
Schematic diagram showing displacement of image points due to the height of a pedestrian.

If we assume that the image film is horizontal, a displacement of the image point caused by the height of the person will be caused as shown in Fig. 5, and Eq. (6) can be applied based on the principle of similar triangles.

(6) $$\eqalign{& \Delta h/R = h/(H - h) \cr & R/(H - h) = r/f \cr & f{\rm / }H = {\delta _h}/\Delta h}$$

where *$\Delta$h* is the differential projection on the ground, *h* is the height of the pedestrian, *H* is the flight height of the UAV, *R* is the horizontal distance from the ground point to the ground nadir point, *r* is the image distance from the projection point of the pedestrian in the image to the image centre point (*r* is positive when the projection point is on the upper side of the image centre point and negative when the projection point is on the lower side), *f* is the focal length, and ${\delta_h}$ is the displacement of the image point caused by the height of the pedestrian.

Equation (7) can be derived from Eq. (6):

(7) $${\delta _h} = r \cdot h/H$$

where ${\delta_h}$ is the displacement of the image point caused by the height of the pedestrian, *r* is the distance in the image from the projection point of the pedestrian in the image to the image centre point, *h* is the height of the pedestrian, and *H* is the flight height of the UAV.

The speed correction can then be calculated from Eq. (8) as follows:

(8) $${v_c} = ({\delta _2}{\rm - }{\delta _1})/n \cdot fps$$

where *v*_*c*_ is the speed correction value (in pixel/s), *n* is the number of frames captured of the pedestrian, *fps* is the frame rate, *δ*_*1*_ is the image point displacement at the starting point, and *δ*_*2*_ is the image point displacement at the end point. Likewise, the speed correction value can be obtained according to Eqs. (3) and (4), as shown in Eq. (9):

(9) $${v_c} = ({r_2}{\rm - }{r_1}) \cdot h \cdot fps \cdot L/n/H/M$$

where *v*_*c*_ is the speed correction value (in m/s), *r*_*1*_ is the image distance from the projection point of the starting point to the image centre point, *r*_*2*_ is the image distance from the projection point of the end point to the image centre point, *n* is the number of frames captured of the pedestrian, *fps* is the frame rate, *H* is the flight height of the UAV, *L* is the length of the field of view, and *M* is the length of the field of the image.

In a two-dimensional plane, if the direction of flight of the drone is not parallel to the direction of movement of the pedestrian, there will be a certain angle between these two directions. Taking the centre point of the image as the origin of coordinates, this angle can be calculated using Eq. (10):

(10) $$\theta = {\rm arctan(}\left| {x - {x_0}} \right|/\left| {y - {y_0}} \right|)$$

where $\theta$ is the angle between the direction of flight and the pedestrian’s direction of walking, (*x*_*0*_, *y*_*0*_) is the centre point of the image, and (*x*, *y*) is the position of the pedestrian in the image.

The speed of the pedestrian after correction is shown in Eq. (11):

(11) $$(v_c)={(r_2-r_1)} \cdot {h} \cdot {fps} \cdot {L} \cdot {\rm cos \theta}/{n}/{H}/{M}$$

where *v*_*c*_ is the pedestrian’s speed (in m/s). This equation shows that the speed correction is related to the relative speed of UAV and the pedestrian (as reflected by the value of *r*), the height of the pedestrian, the height of the UAV, the angle between the pedestrian’s direction of walking and the UAV heading, and certain camera parameters (video conversion frame rate, field of view and focal length of the sensor).

## Results

### Pilot tests

**Figure 6:**
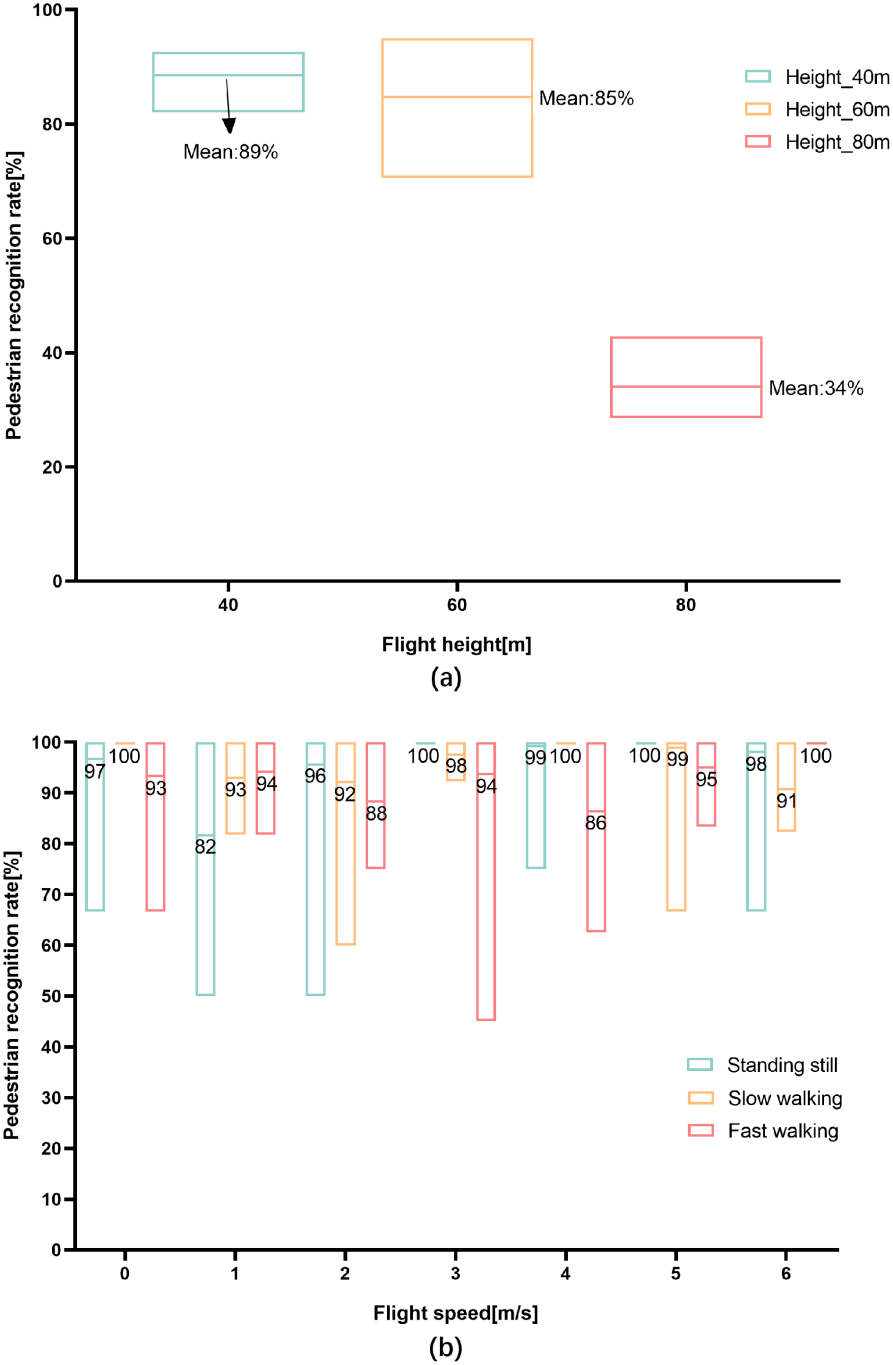
Pedestrian recognition rates at each flight altitude and speed. (A) Pedestrian recognition rates at each flight altitude (*N* = 111); (B) pedestrian recognition rates at each flight speed (three pedestrian states, *N* = 120).

The results of the pilot tests are shown in Fig. 6. The pedestrian recognition rates at three flight altitudes are shown in Fig. 6A, and it can be seen that the overall trend in the pedestrian recognition rate decreased with an increase in flight altitude. A one-way ANOVA indicated significant differences in the pedestrian recognition rates at the three heights (*P* = 0.000 < 0.05). The optimal result was obtained at an altitude of 40 m, with an average recognition rate of 89%. At a height of 60 m, the average recognition rate was reduced to 85%, while the lowest average pedestrian recognition rate was obtained at 80 m, at only 34%. Based on these results, 40 m was determined to be the best flight height, and was used in the data acquisition process in our study area.

Figure 6B illustrates the pedestrian recognition rate obtained from a UAV hovering at the optimal height (40 m) and travelling at different flight speeds for pedestrians with varying walking states. The highest average recognition rate was 100%, while the lowest was 82%. This shows that the recognition rate was affected by both the flight speed of the UAV and the walking state of the pedestrian. Although a two-way ANOVA showed significant differences in pedestrian recognition rates at different flight speeds and pedestrian states (*P* = 0.000 < 0.05), the recognition rates were all higher than the threshold (80%) set for the extraction of walking speeds. According to the results, all seven flight speeds met the standard, meaning that the optimal flight speed was 6 m/s or less, and a speed of 3 m/s was chosen for data collection in the study area.

### Speed error before and after correction

There is a systematic bias between the pedestrian speed extracted from the UAV video and measured on the ground, since the object of observation (the pedestrian) is not always located directly below the UAV and hence the centre of recognition is not always at the pedestrian’s feet. Table 2 shows the average speed errors before and after correction. The average error between the extracted value and the measured value on the ground before correction increases gradually as the UAV speed increases, while there is no such trend after correction. In addition, most average errors become smaller compared to before correction; the maximum average error before correction reaches 0.065 m/s, but after correction this is only 0.010 m/s.

**Table 2:** Average errors in walking speeds before and after speed correction.

Flight speed (m/s)	Standing still (m/s)		Walking slowly (m/s)		Walking fast (m/s)	
	Before correction	After correction	Before correction	After correction	Before correction	After correction
0	0.010	0.010	0.003	−0.013	0.006	0.008
1	0.012	−0.015	0.007	0.001	0.006	0.009
2	0.018	−0.008	0.019	−0.005	0.026	0.010
3	0.021	−0.028	0.021	−0.034	0.026	0.003
4	0.049	−0.018	0.029	−0.036	0.055	−0.002
5	0.046	−0.013	0.042	−0.050	0.055	−0.027
6	0.049	−0.024	0.052	−0.038	0.065	−0.024

**Figure 7:**
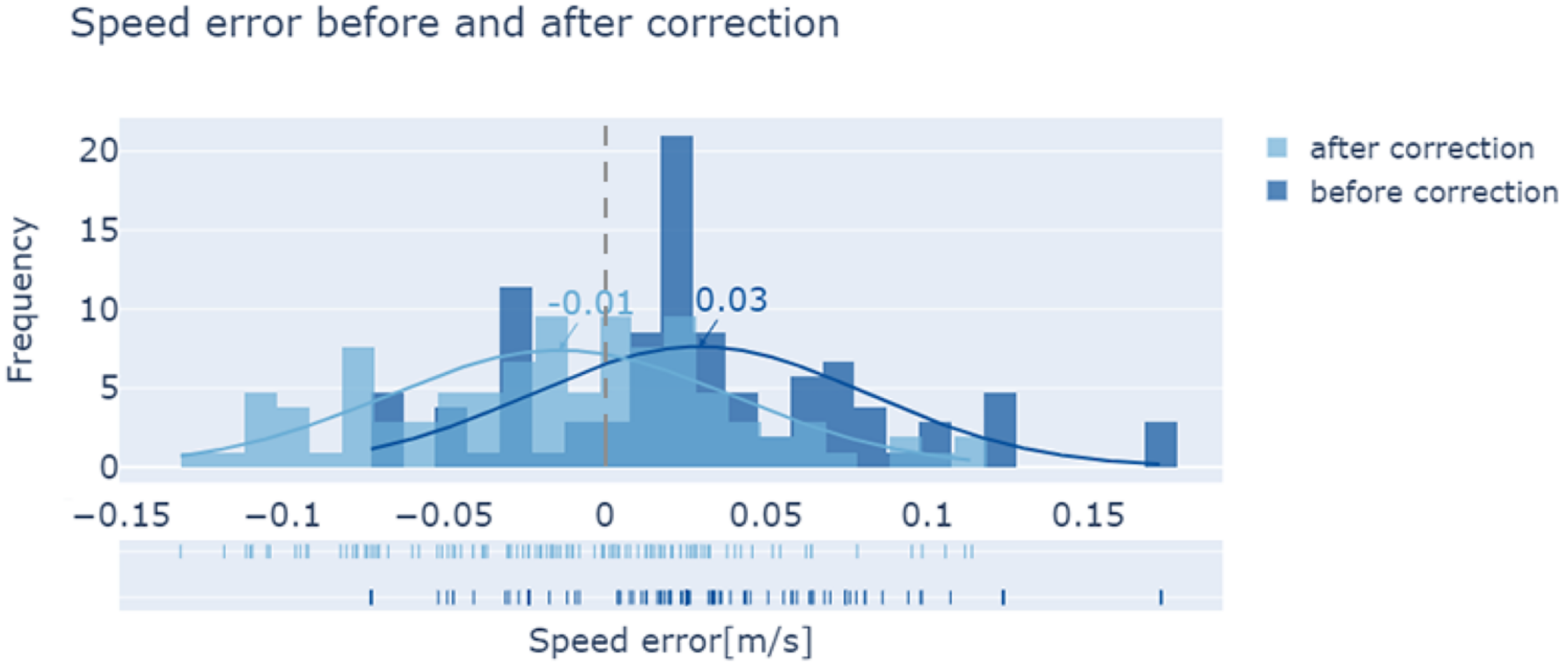
Errors in pedestrian speeds before and after correction.

Figure 7 shows a distribution histogram of the errors before and after correction, which allows for a further comparison. Before speed correction, the average value of the error is 0.03 m/s, and the maximum absolute error is 0.17 m/s. After speed correction, the overall trend in the speed error shows a decrease. The average value of the error is reduced to −0.01 m/s, and the maximum value of the absolute error is 0.13 m/s.

### Walking speeds along a commercial street

**Figure 8:**
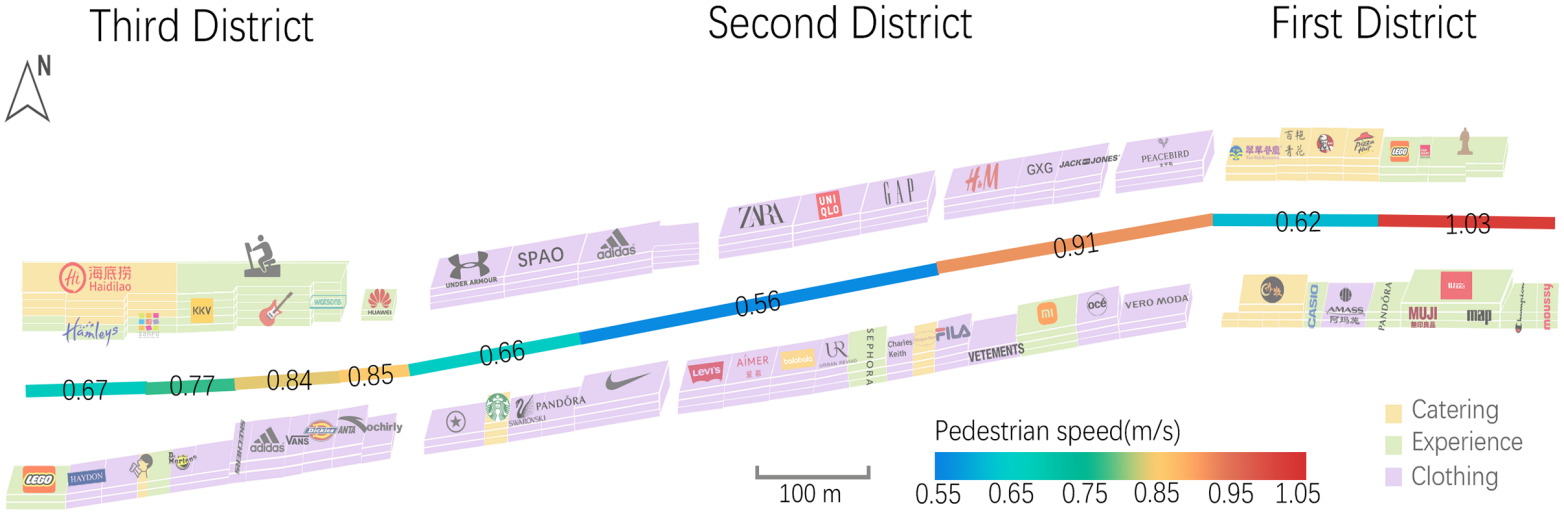
Pedestrian walking speeds extracted in Hanjie Street.

We applied our proposed method to our study area of a commercial street as an application to verify the validity of the method. The results for the pedestrian walking speed are shown in Fig. 8. In general, the speeds for the whole street exhibited spatial heterogeneity, with different speeds in the nine different segments, but the variation between speeds is low for a given segment. The highest walking speed of 1.03 m/s was found for the first district, a section containing ‘experience’ stores such as Lego and Madame Tussauds, while the lowest speed of 0.56 m/s was found for the second district, which is dominated by clothing stores, and contains a cosmetic store and an ice cream store. In addition, for the second district, both of the first two segments had generally low walking speeds, while the third segment showed a significant increase, with a walking speed of 0.91 m/s.

**Figure 9:**
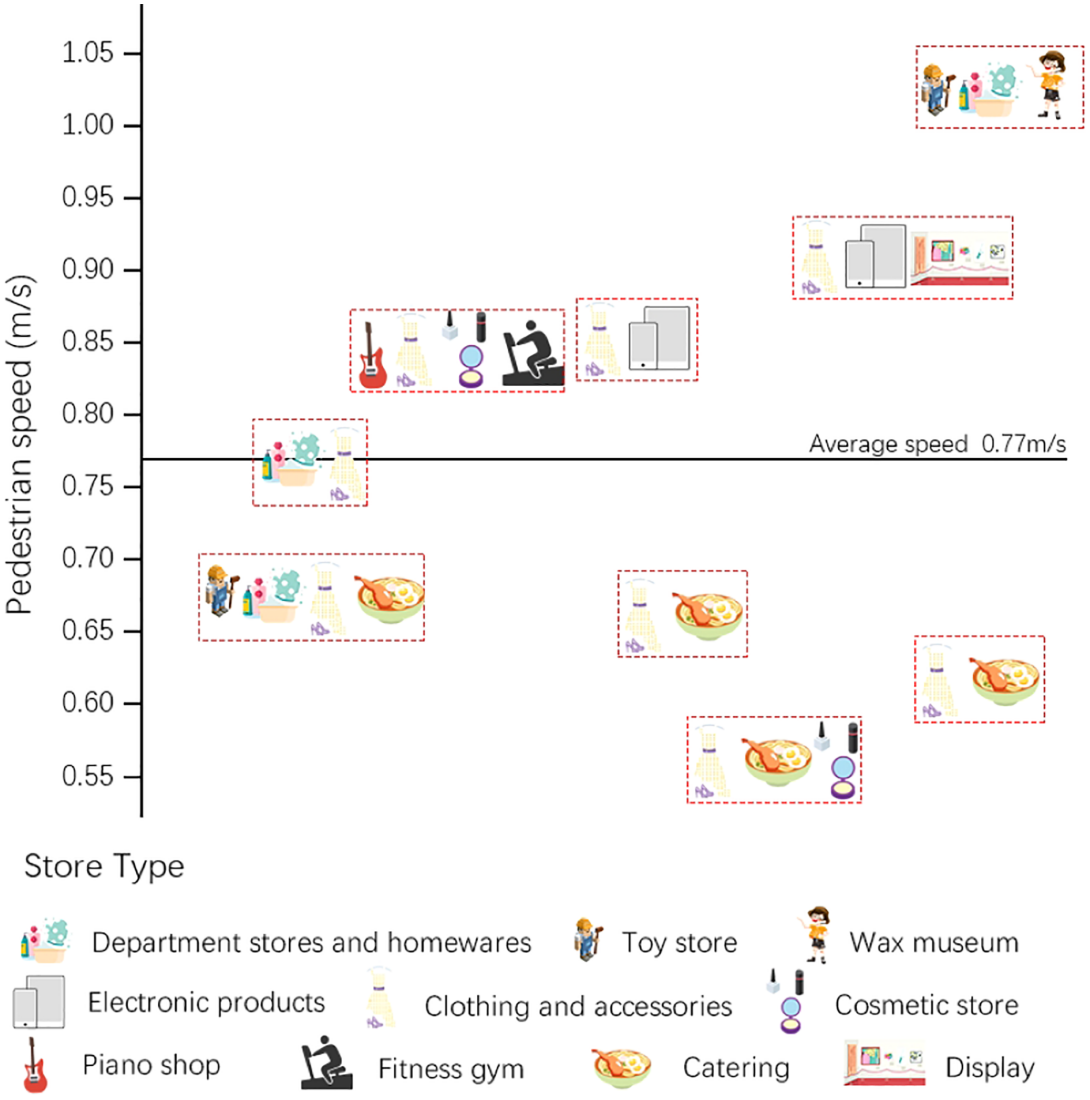
Types of stores on Hanjie Street that affected pedestrian walking speeds.

To confirm that the type of stores on the street had an impact on the walking speeds of the passing pedestrians, we identified the types of store in each of the nine segments, as shown in Fig. 9. The average walking speed over these nine segments was 0.77 m/s, with four segments each showing speeds greater and less than the average. It was found that the slow walking pace exhibited association with street-side snack bars and food restaurants. In addition, walking speeds were faster near stores selling electronic products; this may be due to the fact that more male customers are interested in electronic devices, and according to existing research (Tolea et al., 2010), men generally walk faster. The Wax Museum was another place in which pedestrian speeds were raised, while certain stores did not show a significant effect on pedestrian walking speeds, such as clothing and accessories, department stores and homewares and toy stores.

## Discussion

As an exploratory attempt to extract the walking speeds of pedestrians using a UAV, the results obtained from our experiment require discussion and reflection.

### Optimal flight altitude

In the pilot test carried out to determine the optimal flight altitude, we found that the recognition rate of pedestrians tended to decrease as the flight altitude increased. The average recognition rate of pedestrians was 89% at 40 m, and only 34% at 80 m. The clarity of acquisition of the pedestrians by the UAV camera decreases with an increase in the flight altitude for constant values of the other flight parameters. In addition, when the altitude increases, the pixel size of the pedestrians in the image decreases, and the detection of small targets by deep learning tends to have a lower recognition rate. However, this does not mean that we cannot obtain high pedestrian recognition rates at altitudes of 80 m or even higher. In fact, our dataset for the pilot test was small (only 720 images), while in most cases, if the dataset is large and well labelled, model training can be effectively improved and high target recognition rates can be achieved with no changes to the models or training settings (Jocher, 2021) (according to a view posted on GitHub by the author of YOLOv5).

### Accuracy of corrected walking speed

Although a large number of experiments have been conducted to measure walking speeds, the comparability between existing measurements and our results is low, as walking speed is influenced by numerous parameters and there is no standardisation of measurement conditions (Bosina & Weidmann, 2017). However, based on a comparison of our corrected pedestrian speeds with the measured speeds on the ground, we found that 63.8% of the corrected speeds had an absolute error of below 0.05 m/s, while 90.5% had an absolute error of below 0.1 m/s, and the maximum absolute error was 0.13 m/s. It is clear that the use of this correction means our method has high feasibility and overall accuracy.

**Figure 10:**
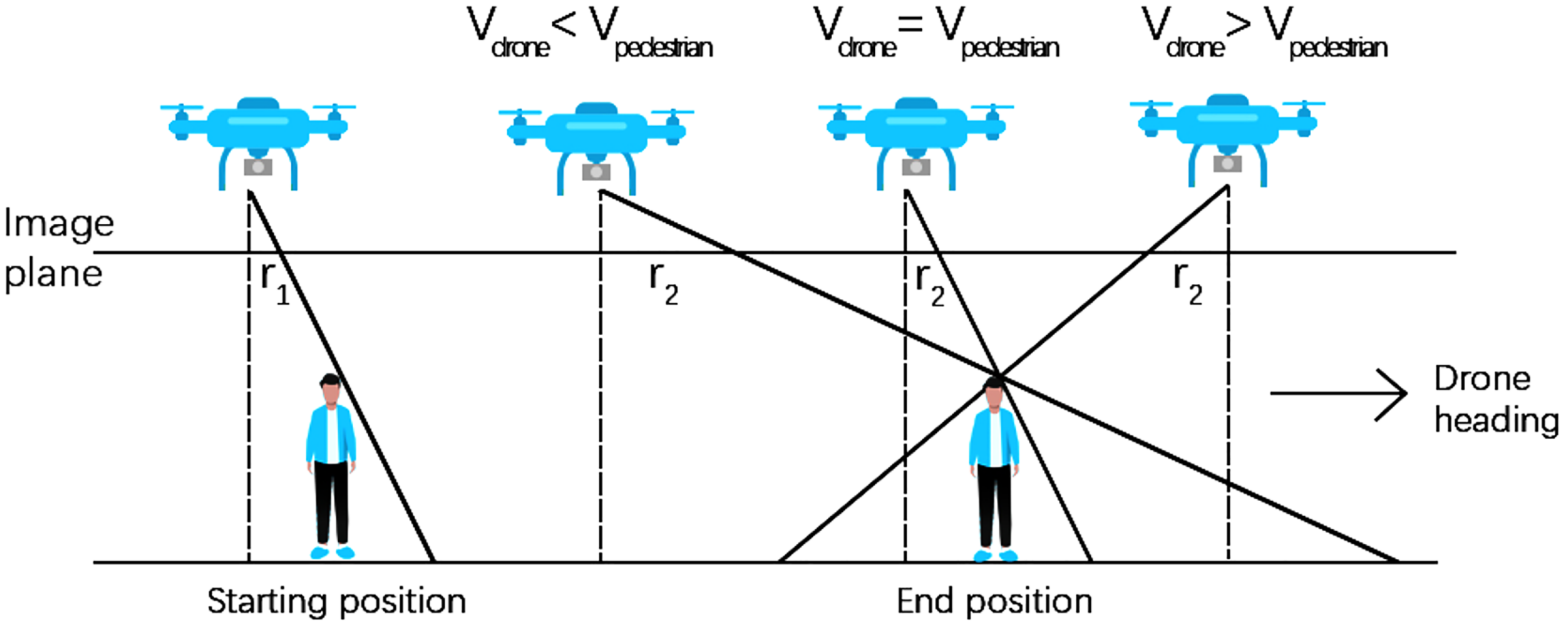
Measurement errors for various relative speed states of the pedestrian and UAV.

As shown in Table 2, the overall error in the extracted pedestrian speeds increases gradually with the UAV speed. According to the principle of central projection, the height of the pedestrian creates an offset in the image compared to the actual position. In Fig. 10, this offset is simplified to *r*, *r*_*1*_ for the offset at the starting position of the pedestrian and *r*_*2*_ for the offset at the end position of the pedestrian. The position of the solid black line intersecting the image plane indicates the position of the pedestrian on the image, while the position of the dotted black line intersecting the image plane is the centre point of the image. When the pedestrian and the drone are moving in the same direction, there are three scenarios for the relative speed of drone and pedestrians. If the drone speed is smaller than the pedestrian speed, the offset increases the distance moved by the pedestrian in the image in the two frames before and after. If the two speeds are equal, the offset has no effect to the distance. If the drone speed is greater than the pedestrian speed, the offset decreases the distance. In the pilot test, the UAV moved in the same direction as the pedestrians, and the flight speed was typically higher than the pedestrian walking speeds. According to Eq. (1), as the distance moved by the pedestrian in the image decreases, the real distance increases, leading to a higher calculated speed. The higher the speed of the UAV compared with the pedestrian, the longer the distance moved by the pedestrian in the image will become, resulting in a larger error in the speed.

To eliminate the displacement in the image points caused by the central projection, we apply a speed correction. Although we describe it here for the case where the drone and pedestrians are moving in the same direction, our speed correction formula can also be applied to the case where the UAV and pedestrians are moving in the opposite direction (Eq. (6)). In addition, as can be seen in Table 2, when pedestrians walk fast or UAVs fly fast, it generally causes more difficulties in pedestrian recognition (it can be seen from the absolute error before correction), but the correction can well correct these samples with larger deviations. In other words, the correction can be well adapted to the difficult samples and improve the overall computational accuracy.

### Reduce the false detection rate

The issue of how to reduce the false detection rate is also a significant problem. Because our study area is a pedestrianised street, no cyclists appeared in the captured video data. However, we found in the relevant trials that interference can easily arise from cyclists (bicycles or electric bikes). We tried to divide the people into walkers and cyclists at the labelling stage, and eventually most of the walkers were correctly distinguished, but a small fraction of the cyclists was still mis-detected. this is a problem worth considering when this method is to be applied to neighbourhoods with mixed pedestrian and bicycle traffic.

There are two ways to solve this problem: the first is to ignore the classification of cyclists when training the model, as there will be a clear double-peak pattern in the speed distribution histogram plots that represents cyclists and pedestrians. A threshold can then be set to distinguish between them. In the second approach, the false detection rate can be reduced by adding negative samples, cropping the region of the original image containing only cyclists for use as negative samples, and using these and the positive samples together to train the model.

### Limitations and future work

Finally, our method still has some limitations that need to be overcome. During the pedestrian recognition process, a large number of annotated datasets are required for model training, which imposes a high labour cost. Although many public pedestrian datasets exist for use, they either do not have annotated labels or were not captured from an overhead view, meaning that the precision of speed extraction cannot be guaranteed. Although it is easy to capture numerous pedestrian images using a UAV, manual annotation is required, and this is a problem that is also studied in deep learning.

We also found some interesting things from extracting walking speeds along Hanjie Street. As shown in Fig. 8, there were clear differences in the walking speeds along the different subdivisions of this street, which may be related to the category, brand and decoration of the stores, or possibly to the season or the time of video collection. In addition, it has been shown that pedestrian density also has an effect on walking speed (Franěk, 2013; Minegishi, 2021). Our current study does not consider the effect of pedestrian density on walking speed for the time being. In future work, we will incorporate more influencing factors to further explore the correlations between street environments and pedestrian speeds.

## Conclusions

Unlike existing research methods, such as those based on timing gates and wearable devices, our method can collect the walking speeds of numerous pedestrians within a large area without disturbing them. It is also less expensive and does not require the placement of measurement facilities. If high-quality video data can be collected using drones, it is possible to extract the walking speeds of pedestrians on city streets. Real-time measurements of walking speeds on urban streets can help us to gain a deeper understanding of how the settings and layouts of urban spaces work for people, which is one of the core issues of urban geography.

## Supplemental Information

10.7717/peerj-cs.1226/supp-1 Supplemental Information 1 a photo of the street taken by a drone.

## Additional Information and Declarations

### Data Availability

The following information was supplied regarding data availability: The code and raw data are available at figshare: jiao, dan (2022): walking speed dataset. figshare. Dataset. https://doi.org/10.6084/m9.figshare.21435252.v2.

## References

[ref-1] Al-Azzawi M, Raeside R (2007). Modeling pedestrian walking speeds on sidewalks. Journal of Urban Planning and Development.

[ref-2] Bian C, Yang Z, Zhang T, Xiong H (2016). Pedestrian tracking from an unmanned aerial vehicle.

[ref-3] Bosina E, Weidmann U (2017). Estimating pedestrian speed using aggregated literature data. Physica A: Statistical Mechanics and its Applications.

[ref-4] Bouafif H, Kamoun F, Iqbal F, Marrington A (2018). Drone forensics: challenges and new insights.

[ref-5] Cao Z-Q, Sai B, Lu X (2020). Review of pedestrian tracking: algorithms and applications. Acta Physica Sinica.

[ref-6] Cha M, Han S, Kim H, Mun D (2017). User-driven treadmill using walking speed estimated from plantar pressure sensor. Electronics Letters.

[ref-7] Chandra S, Bharti AK (2013). Speed distribution curves for pedestrians during walking and crossing. Procedia-Social and Behavioral Sciences.

[ref-8] Chang Y-C, Chen H-T, Chuang J-H, Liao I-C (2018). Pedestrian detection in aerial images using vanishing point transformation and deep learning.

[ref-9] Chen Z, Zhao X, Shi R (2016). Walking speed modeling on transfer passengers in subway passages.

[ref-10] Ciaparrone G, Sánchez FL, Tabik S, Troiano L, Tagliaferri R, Herrera F (2020). Deep learning in video multi-object tracking: a survey. Neurocomputing.

[ref-11] Derpanis KG (2010). Overview of the RANSAC Algorithm. Image Rochester NY.

[ref-12] Duim E, Lebrão ML, Antunes JLF (2017). Walking speed of older people and pedestrian crossing time. Journal of Transport & Health.

[ref-13] Fang Z, Kim P (2020). Comparison of deep-learning algorithms for the detection of railroad pedestrians. Journal of Information and Communication Convergence Engineering.

[ref-14] Finnis KK, Walton D (2008). Field observations to determine the influence of population size, location and individual factors on pedestrian walking speeds. Ergonomics.

[ref-15] Fischler MA, Bolles RC (1981). Random sample consensus: a paradigm for model fitting with applications to image analysis and automated cartography. Communications of the ACM.

[ref-16] Franěk M (2013). Environmental factors influencing pedestrian walking speed. Perceptual and Motor Skills.

[ref-17] Franěk M, Režnỳ L (2021). Environmental features influence walking speed: the effect of urban greenery. Land.

[ref-18] Fritz S, Lusardi M (2009). White paper: walking speed: the sixth vital sign. Journal of Geriatric Physical Therapy.

[ref-19] Gore N, Dave S, Shah J, Jain M, Rathva D, Garg V, Mathew T, Joshi G, Velaga N, Arkatkar S (2020). Comparative analysis of pedestrian walking speed on sidewalk and carriageway. Transportation Research.

[ref-20] Hediyeh H, Sayed T, Zaki MH, Mori G (2014). Pedestrian gait analysis using automated computer vision techniques. Transportmetrica A: Transport Science.

[ref-21] Hussein M, Sayed T, Reyad P, Kim L (2015). Automated pedestrian safety analysis at a signalized intersection in New York City: automated data extraction for safety diagnosis and behavioral study. Transportation Research Record.

[ref-22] Jin C-J, Shi X, Hui T, Li D, Ma K (2021). The automatic detection of pedestrians under the high-density conditions by deep learning techniques. Journal of Advanced Transportation.

[ref-23] Jocher G (2020). YOLOv5 Docs for full documentation on training, testing and deployment. GitHub.

[ref-24] Jocher G (2021). View posted on GitHub by the author of YOLOv5 about how to increase recall. GitHub.

[ref-25] Kim D (2020). Pedestrian and bicycle volume data collection using drone technology. Journal of Urban Technology.

[ref-26] Kong PW, Chua YHK (2014). Start-up time and walking speed in older adults under loaded conditions during simulated road crossing. Experimental Aging Research.

[ref-27] Lan W, Dang J, Wang Y, Wang S (2018). Pedestrian detection based on YOLO network model.

[ref-28] Li H, Wu Z, Zhang J (2016). Pedestrian detection based on deep learning model.

[ref-29] Liang S, Leng H, Yuan Q, Wang B, Yuan C (2020). How does weather and climate affect pedestrian walking speed during cool and cold seasons in severely cold areas?. Building and Environment.

[ref-30] Lowe DG (1999). Object recognition from local scale-invariant features.

[ref-31] Lowe DG (2004). Distinctive image features from scale-invariant keypoints. International Journal of Computer Vision.

[ref-32] Lusardi MM (2012). Is walking speed a vital sign? Absolutely!. Topics in Geriatric Rehabilitation.

[ref-33] Martin E, Kim S, Unfried A, Delcambre S, Sanders N, Bischoff B, Saavedra R (2019). 6th vital sign app: testing validity and reliability for measuring gait speed. Gait & Posture.

[ref-34] MejiaCruz Y, Franco J, Hainline G, Fritz S, Jiang Z, Caicedo JM, Davis B, Hirth V (2021). Walking speed measurement technology: a review. Current Geriatrics Reports.

[ref-35] Mekala SH, Baig Z (2019). Digital forensics for drone data-intelligent clustering using self organising maps.

[ref-36] Middleton A, Fritz SL, Lusardi M (2015). Walking speed: the functional vital sign. Journal of Aging and Physical Activity.

[ref-37] Minegishi Y (2021). Experimental study of the walking behavior of crowds mixed with slow-speed pedestrians as an introductory study of elderly-mixed evacuation crowds. Fire Safety Journal.

[ref-38] Miyazato T, Uehara W, Nagayama I (2019). Development of a free viewpoint pedestrian recognition system using deep learning for multipurpose flying drone. Electronics and Communications in Japan.

[ref-39] Montero-Odasso M, Sarquis-Adamson Y, Kamkar N, Pieruccini-Faria F, Bray N, Cullen S, Mahon J, Titus J, Camicioli R, Borrie MJ, Bherer L, Speechley M (2020). Dual-task gait speed assessments with an electronic walkway and a stopwatch in older adults. A reliability study. Experimental Gerontology.

[ref-40] Montufar J, Arango J, Porter M, Nakagawa S (2007). Pedestrians’ normal walking speed and speed when crossing a street. Transportation Research Record: Journal of the Transportation Research Board.

[ref-41] Morey M, Ryan S, Kelly J, Liu C, Hawkins K, Schwartz K, Bettger JP (2017). The 6th vital sign: a mobile app for population health surveillance of walking speed. Innovation in Aging.

[ref-42] Nolan CM, Kon SS, Patel S, Jones SE, Barker RE, Polkey MI, Maddocks M, Man WD (2018). Gait speed and pedestrian crossings in COPD. Thorax.

[ref-43] Obuchi SP, Kawai H, Murakawa K (2020). Reference value on daily living walking parameters among Japanese adults. Geriatrics & Gerontology International.

[ref-44] Oh S-L, Kim DY, Bae JH, Jung H, Lim JY (2019). Comparison of the use of a manual stopwatch and an automatic instrument for measuring 4-m gait speed at the usual walking pace with different starting protocols in older adults. European Geriatric Medicine.

[ref-45] Rastogi R, Thaniarasu I, Chandra S (2011). Design implications of walking speed for pedestrian facilities. Journal of Transportation Engineering.

[ref-46] Redmon J, Divvala S, Girshick R, Farhadi A (2016). You only look once: unified, real-time object detection.

[ref-47] Rădescu R, Dragu M (2019). Automatic analysis of potential hazard events using unmanned aerial vehicles.

[ref-48] Saeidi M, Ahmadi A (2018). Deep learning based on CNN for pedestrian detection: an overview and analysis.

[ref-49] Sikandar T, Rabbi MF, Ghazali KH, Altwijri O, Alqahtani M, Almijalli M, Altayyar S, Ahamed NU (2021). Using a deep learning method and data from two-dimensional (2D) marker-less video-based images for walking speed classification. Sensors.

[ref-50] Silsupadol P, Teja K, Lugade V (2017). Reliability and validity of a smartphone-based assessment of gait parameters across walking speed and smartphone locations: body, bag, belt, hand, and pocket. Gait & Posture.

[ref-51] Silva AMCB, da Cunha JRR, da Silva JPC (2014). Estimation of pedestrian walking speeds on footways. Proceedings of the Institution of Civil Engineers-Municipal Engineer.

[ref-52] Stephan Y, Sutin AR, Terracciano A (2015). Feeling younger, walking faster: subjective age and walking speed in older adults. Age.

[ref-53] Tolea MI, Costa PT, Terracciano A, Griswold M, Simonsick EM, Najjar SS, Scuteri A, Deiana B, Orrù M, Masala M, Uda M, Schlessinger D, Ferrucci L (2010). Sex-specific correlates of walking speed in a wide age-ranged population. Journals of Gerontology Series B: Psychological Sciences and Social Sciences.

[ref-54] van Loo MA, Moseley AM, Bosman JM, de bie RA, Hassett L (2003). Inter-rater reliability and concurrent validity of walking speed measurement after traumatic brain injury. Clinical Rehabilitation.

[ref-55] Wang C, Sun X, Li H (2019). Research on pedestrian tracking algorithm based on deep learning framework. Journal of Physics: Conference Series.

[ref-56] Warden SJ, Kemp AC, Liu Z, Moe SM (2019). Tester and testing procedure influence clinically determined gait speed. Gait & Posture.

[ref-57] Willis A, Gjersoe N, Havard C, Kerridge J, Kukla R (2004). Human movement behaviour in urban spaces: implications for the design and modelling of effective pedestrian environments. Environment and Planning B: Planning and Design.

[ref-58] Wojke N, Bewley A, Paulus D (2017). Simple online and realtime tracking with a deep association metric.

[ref-59] Xiao Y, Zhou K, Cui G, Jia L, Fang Z, Yang X, Xia Q (2021). Deep learning for occluded and multi-scale pedestrian detection: a review. IET Image Processing.

[ref-60] Xue Y, Ju Z (2021). Multiple pedestrian tracking under first-person perspective using deep neural network and social force optimization. Optik.

[ref-61] Yeom S, Cho I-J (2019). Detection and tracking of moving pedestrians with a small unmanned aerial vehicle. Applied Sciences.

[ref-62] Youdas JW, Hollman JH, Aalbers MJ, Ahrenholz HN, Aten RA, Cremers JJ (2006). Agreement between the GAITRite walkway system and a stopwatch-footfall count method for measurement of temporal and spatial gait parameters. Archives of Physical Medicine and Rehabilitation.

