# Supplementary figures and images for: Pedestrian walking speed monitoring at street scale by an in-flight drone
Source: PeerJ Comput Sci. 2023 Jan 25;9:e1226. doi: 10.7717/peerj-cs.1226 (PMC10280686; doi:10.7717/peerj-cs.1226)

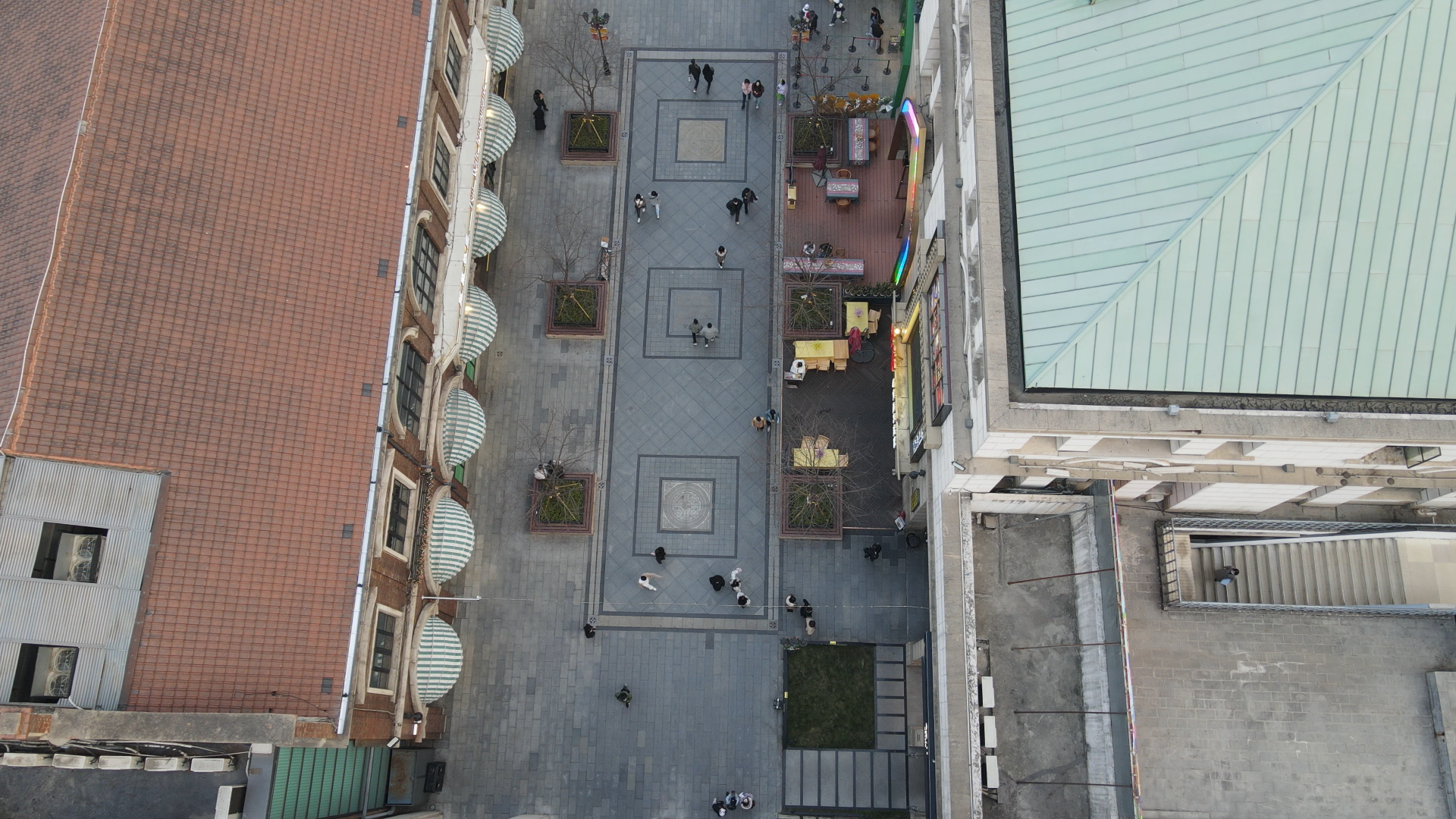

Supplement: Supplemental Information 1 [file peerj-cs-09-1226-s001.png]
